# On the Development of Health-Based Ventilation Guidelines: Principles and Framework

**DOI:** 10.3390/ijerph15071360

**Published:** 2018-06-28

**Authors:** Paolo Carrer, Eduardo de Oliveira Fernandes, Hugo Santos, Otto Hänninen, Stylianos Kephalopoulos, Pawel Wargocki

**Affiliations:** 1 Occupational and Environmental Health, Department of Biomedical and Clinical Sciences “L. Sacco” University of Milan, IT-20157 Milan, Italy; 2 University of Porto, 4200-465 Porto, Portugal; eof@fe.up.pt; 3 INEGI-Institute of Science and Innovation in Mechanical and Industrial Engineering, 4200-465 Porto, Portugal; hsantos@inegi.up.pt; 4 National Institute for Health and Welfare, FI-70701 Kuopio, Finland; otto.hanninen@thl.fi; 5 European Commission, Joint Research Centre, Directorate for Health, Consumers and Reference Materials, 20127 Ispra, Italy; stylianos.kephalopoulos@ec.europa.eu; 6 International Centre for Indoor Environment and Energy, Department of Civil Engineering, Technical University of Denmark, DK-2800 Kongens Lyngby, Denmark; paw@byg.dtu.dk

**Keywords:** indoor air quality, health risks, ventilation, building management

## Abstract

This paper summarizes the results of HealthVent project. It had an aim to develop health-based ventilation guidelines and through this process contribute to advance indoor air quality (IAQ) policies and guidelines. A framework that allows determining ventilation requirements in public and residential buildings based on the health requirements is proposed. The framework is based on three principles: 1. Criteria for permissible concentrations of specific air pollutants set by health authorities have to be respected; 2. Ventilation must be preceded by source control strategies that have been duly adopted to improve IAQ; 3. Base ventilation must always be secured to remove occupant emissions (bio-effluents). The air quality guidelines defined by the World Health Organization (WHO) outside air are used as the reference for determining permissible levels of the indoor air pollutants based on the principle that there is only one air. It is proposed that base ventilation should be set at 4 L/s per person; higher rates are to be used only if WHO guidelines are not followed. Implementation of the framework requires technical guidelines, directives and other legislation. Studies are also needed to examine the effectiveness of the approach and to validate its use. It is estimated that implementing the framework would bring considerable reduction in the burden of disease associated with inadequate IAQ.

## 1. Introduction

### 1.1. Rationale for Developing Health-Based Ventilation Guidelines

Ventilation is one of the strategies that are used for controlling indoor air quality (IAQ) in buildings. It supplies outdoor air that is generally assumed to be clean or cleaned and fresh, to reduce exposure by removing and, ultimately, to dilute the air pollutants indoors. This process is undertaken with the aim of reducing exposures to hazardous pollutants indoors and consequently the associated risks for health, comfort and wellbeing, as well as work performance and learning. That the risks are likely has been documented in numerous studies [1,2,3].

There have been several reviews carried out to date that concluded, at which ventilation rates the risks for health are likely to occur [4,5,6,7,8]. Still, however, the current ventilation standards, which are by and large referred to in building codes, prescribe predominantly the ventilation rates that address the risks associated with discomfort caused by unpleasant odours and due to unacceptable air quality as perceived by visitors to and occupants in the buildings. This is best illustrated by the short summary of the methods used to determine ventilation rates in two best known ventilation standards, the one used in Europe [9] and one that is used in the U.S. [10].

Ventilation rates in ASHRAE 62.1 Standard [10] are estimated using the so-called ventilation rate or IAQ procedures. In the former, the actual ventilation rates to be used in buildings are prescribed by the standard. These rates deal with the pollution load from both people and the load from non-human sources of pollution in different types of spaces with the pre-defined occupancy. They are intended to ensure that the majority of visitors and occupants (80% or more) do not express dissatisfaction with indoor air quality in buildings and are for spaces that are free from environmental tobacco smoke (ETS). In the latter procedure, ventilation rates are calculated based on the contaminants of concern that shall be predetermined during the process of designing ventilation rates. The list of potential contaminants of concern is presented in the informative appendix included in the standard; they are though not formally prescribed by the standard. These contaminants and their concentrations are then compared against the reference values defined by cognizant authorities; information on some of the available guideline values is also included in the appendix in the standard and again this is not a formal standard requirement. Ventilation rates are eventually calculated based on the reference values for pollutants and their assumed emission rates. At the final stage, it must be documented that the majority of people exposed to pollutants of concern should not express dissatisfaction with IAQ as is the case in the ventilation rate procedure. Consequently, even though the IAQ procedure can be considered as being based to some extent on health requirements it is also defining the ventilation rates that are intended to reduce the risks for sensory discomfort. The standard requires that for both procedures the air supplied by ventilation system must be of a high quality. The requirements are given only for filtration with the specific minimum efficiency that must be used in case the levels of PM_10_ and PM_2.5_ in outdoor air do not meet the ambient standards. There are also requirements regarding ozone removal from the supplied air if its concentration is too high; other ambient pollutants are not dealt with in the standard unless considered in the IAQ procedure.

Standard EN 15251 [9] follows principally the same approaches as included in ASHRAE 62.1 [10]. The few important differences are described in the following. It prescribes ventilation rates that assist the designers in achieving three levels of air quality indoors defined as the percentage of dissatisfied visitors entering the space; these levels are set to be 15%, 20% and 30% dissatisfied with the quality of air indoors. The standard does not prescribe ventilation rates for the occupants in buildings. Unlike ASHRAE standard, it also defines different ventilation rates to deal with the non-human sources of pollution depending on their load. For this purpose, the buildings with a very low pollution, with low pollution and other buildings (called non-low polluting) are defined; the appendix being an informative part of the standard provides the guidelines that should be used when determining the building category based on the pollution load. The standard allows calculation of ventilation rates based on the guideline levels for pollutants, as is the case of IAQ procedure in the ASHRAE Standard 62.1 [10]. However, the rates calculated in this way are regularly lower than the rates required to ensure that the percentage of dissatisfied with air quality are not exceeded. 

EN15251 [9] is currently under revision [11]. The draft of revised standard called prEN16798-1 [12] contains similar approaches to determine ventilation rates as included in the EN15251 [9]. The important addition, following the early findings and publications of HealthVent project is that the revision of the standard specifies the ventilation rate of 4 L/s per person as the minimum rate during occupancy once the WHO Guidelines are met. It also clearly states that the control of emission of non-human pollutants shall be the primary strategy for maintaining acceptable air quality. This falls in line with principles of the framework for determining health-based ventilation guidelines proposed by HealthVent project and described later.

It is important to recall and underline that the rates prescribed in ASHRAE 62.1 [10] and by EN 15251 [9] originate from studies that investigated the effects of indoor air pollutants on the quality of air as it is perceived by the visitors to a space or the occupants in a space (i.e., respectively by individuals whose olfactory system is not adapted to pollutants that can be perceived by olfactory system). These rates were determined using the results of studies performed in the laboratory and in the field when the air was polluted by human bioeffluents, building-related pollution, that is, other sources of pollution in a building excluding human bioeffluents and both human bioeffluents and building [13,14,15,16,17]. The rates prescribed by the mentioned standards are thus based primarily on the sensory perception of quality of air. It is implicitly assumed that these rates would create protection against other risks including the risks for health but are not determined directly to deal with health effects due to exposures to air pollutants. 

It is subsequently fair to say that none of the mentioned standards presents a coherent, clear and consistent strategy on how to design ventilation rates that refer to and respect directly health requirements, although some procedures for addressing health risks are suggested. The guidelines for defining health-based ventilation rates in a systematic way are consequently required. Such guidelines, when developed, should ensure that building occupants would be properly protected against potential health risks caused by exposures to hazardous air pollutants. 

The need for health-based ventilation guidelines arises also from the European health policies which are included in both the EU Environment and Health Action Plan 2004–2010 and the EU Public Health Program 2007–2013. These documents give high priority to reducing the burden of disease (BoD) due to inadequate air quality. The health consequences resulting from inadequate indoor air quality expressed as BoD were estimated by two European projects, EnVIE [18] and IAIAQ [19]. They linked health effects to exposure to indoor air pollutants. The projects estimated that ca. 2 million disability-adjusted life years (DALYs) are lost annually for the population of 26 European countries because of exposure to pollutants indoors. This BoD results in considerable societal costs assuming that one DALY is worth about €115,000 [20]. For example, the socio-economic costs of indoor air pollution in France were estimated to reach €20 billion [21]. It is obvious that such costs must not be neglected.

The EnVIE and IAIAQ projects estimated further that control of indoor and outdoor sources of pollution, including moisture, could reduce the BoD from inadequate indoor air quality by approx. 0.7 million DALYs in 26 European countries and that mandating the regular inspection and maintenance of ventilation and air conditioning systems in all buildings would reduce this BoD by an additional 0.2 million DALYs. They recommended therefore that health-based ventilation guidelines should be developed as it would bring considerable reduction of the BoD due to poor IAQ. 

The development of health-based ventilation guidelines is in line with the World Health Organization’s (WHO) declaration [22] which advocates that every citizen has the right to good IAQ that does not endanger health and that growing up and living in healthy environments is a basic Human Right. Although it is implicit that buildings should follow this declaration, they often fail to perform as designed, thereby increasing the health risks experienced by their occupants [23].

### 1.2. Objective

Taking the above evidence and arguments into consideration, it seemed timely to propose a solid foundation for ventilation integrating its role in exposure control and achieving IAQ by addressing explicitly health criteria. To address this call, the project “Health-Based Ventilation Guidelines for Europe” (HealthVent) was initiated. It was supported by grants from the European Commission Directorate General for Health and Consumers under the Second Programme of Community Action in the Field of Health (2008–2013). The main objective of HealthVent projects was to propose the framework for setting health-based ventilation guidelines, estimate the benefits if its application and list the policies that will support its implementation in the European context. The present paper describes the main outcomes of this assignment, whereas the detailed account of the project’s finding can be found in the ECA Report 30 [24].

## 2. Proposed Framework for Setting Health-Based Ventilation Guidelines

### 2.1. Terms and Conditions for Developing the Guidelines

Before developing the framework for setting health-based ventilation guidelines it was acknowledged that ventilation is an important and prevailing strategy for controlling IAQ but it is not the only strategy that is available. Consequently, when developing health-based ventilation guidelines, a holistic approach to the relevant built environment was considered, taking into account different strategies for controlling IAQ and examining not only the building but also its surroundings. To address the former, it was acknowledged that the guidelines should admit the dependence and interrelationship between ventilation and other strategies for controlling IAQ, such as source control, local exhaust (point exhaust), filtration and air cleaning. To address the latter, it was admitted that the outdoor environment and outdoor air quality should be considered as well whether it is the street and the surroundings and whether it is urban or rural. 

The above approach takes into account that the IAQ of a given generic indoor space is the result of interactions between the outdoor air, the building itself and the system that provides ventilation. In this simplified construct, outdoor air is regarded as the air around the building that is introduced into the building either by natural or mechanical means. The building is the enclosure with a specific indoor air or a cluster of several interconnected enclosures and spaces with their own indoor air dynamics, including the relationships between these and the outdoor air that enters through openings such as windows, doors or other openings, driven by natural forces. These openings can be partially or fully open during the day, depending on the local climate and weather conditions. Finally, the ventilation system is understood as an additional technical system, device or equipment installed in the building to control, whenever needed, the quantity and the quality of the outdoor air brought into the building or into a specific indoor space. The presented construct support clearly the statement that there is ‘only one air’ and thus that the air around the buildings, passing through the structure and the system and the air indoors is simply one continuum that should be subjected to the same regulations and requirements as it has been already postulated by WHO declaration [22]. The interaction between outdoor air, building and the ventilation system was taken into account when guidelines were developed and guidelines could not address one of them in isolation.

Besides the above considerations, it was proposed that the construction of health-based ventilation guidelines should comply with the following three principles:

(1) The guidelines should refer to established exposure guidelines that reduce health risks.
(2) The guidelines should acknowledge that ventilation is a subordinate strategy for improving IAQ after the adoption of the due air pollution source control measures.
(3) The guidelines should define the “base ventilation rate” that must always be guaranteed to remove emissions from humans (human bio-effluents) and, ultimately, the criteria to select the specific “health-based ventilation rate” for a specific building.

It is worth to note that these considerations follow the general procedures for developing IAQ guidelines as discussed among others by Seifert et al. [25].

#### 2.1.1. Health-Based Exposure Guidelines and Exposure Limits

As argued above, health-based ventilation guidelines need to refer to health-based exposure limit guidelines. There are actually several guidelines and regulations that define exposure limits that reduce health risks. Most of them, such as the limits of the Occupational Safety and Health Association (OSHA) and the Threshold Limit Values (TLVs) of the American Conference of Governmental Industrial Hygienists (ACGIH), have been specifically developed to protect against health risks in occupational settings. They are thus not directly applicable to non-industrial indoor environments. 

On the other hand, the World Health Organization (WHO) has since 1987 been publishing guidelines for air quality that deal with protection against health risks for the general population [26,27,28,29,30]. It is consequently reasonable to assume that the WHO guidelines create a more adequate and relevant reference for exposure limits for non-industrial environments than exposure limits that were set specifically for industrial environments. While some of the WHO air quality guidelines were at first defined as requirements for outdoor air [26,28], it must be recalled that their scope was always intended to apply also to indoor air, under the “one air” concept. In adopting the holistic approach for managing exposure indoors, these guidelines must be followed indoors because many pollutants associated with outdoor air are also present indoors, as they enter buildings through windows and doors, mainly in the EU Southern belt countries, as well as through cracks, by infiltration, or due to ventilation or generic “airing out,” respecting the construct described above connecting outdoor environment with indoor conditions.

To extend the existing air quality guidelines [26,28], the WHO issued specific guidelines in 2010 for indoor air quality regarding nine specific pollutants: carbon monoxide, nitrogen dioxide, benzene, trichloroethylene, tetrachloroethylene, formaldehyde, naphthalene, polycyclic aromatic hydrocarbons and radon [29]. These guidelines used recommendations made by the EU-INDEX project [31,32] that was carried out to support the definition of health-based exposure limits for air quality in Europe (Table 1). The intention of those guidelines was to reduce the health risks associated with exposure to the listed pollutants and to provide a scientific basis for legally enforceable standards in all regions of the world. The substances included in these guidelines are common indoor pollutants but just a few of the many hundreds of chemicals that can be identified indoors, that is, the list is not exhaustive. For example, it does not include airborne particulate matter (PM). However, during the 2005 update of WHO’s air quality guidelines [28] it was concluded that as long there is no convincing evidence for a difference in the hazardous nature of PM from indoor sources as compared to those from outdoor sources, the general recommendations for PM are also applicable to indoor spaces. 

In addition to the above-mentioned air quality guidelines, WHO published in 2009 the guidelines on dampness and mould [29]. These guidelines state that persistent dampness and microbial growth on interior surfaces and in building structures should be avoided or minimized. They also state that there are no well-established relationships between dampness, microbial exposure and any quantifiable health effects or for acceptable levels of contamination by microorganisms.

WHO air quality guidelines summarized in Table 1 should thus be recognized to provide currently the most rational, coherent, scientifically sound and consistent reference for the health risks associated with exposure to air pollutants. They were consequently selected as the reference for determining acceptable exposure levels when developing health-based ventilation guidelines.

#### 2.1.2. Ventilation as a Subordinate Strategy for Controlling IAQ

As argued above, health-based ventilation guidelines need to consider other methods for controlling IAQ and not only ventilation. It has long been common practice to use general ventilation as an energy carrier for heating/cooling indoors based on criteria where ‘added on’ systems were seen as instruments for correction of indoor climatic factors not foreseen and tackled at the building design stage. To this end, ventilation became a panacea to cope with indoor air humidity and temperature as well as with air pollution and consequently other methods for controlling IAQ such as source control, filtration and air cleaning, or local (point) exhaust devices for entrapment of pollutants at source have not been properly acknowledged. Principally and from the risk management perspective, source control appears to be more rational and effective when applied sensibly and for specific types of sources than dilution of indoor air by ventilation. It is fair to say that source control is the most efficient strategy for controlling IAQ, supporting ventilation by reducing the initial exposures to pollutants that originate indoors. There are several excellent examples of the efficiency of source control including promotion of low-emitting materials in construction, decoration and furnishings and management of activities that lead to increased pollution indoors such as the ban on smoking. Regardless, it must also be admitted that source control has not received sufficient recognition by considering it as the primary strategy for controlling IAQ similarly as has been done for outdoor air quality control. 

To admit the prime position of source control to achieve high IAQ, the position was taken when developing health-based ventilation guidelines that ventilation is subordinate (complementary) to source control and never the reverse. It was thus accepted that health-based ventilation can be defined only when all other means of controlling pollution have been fully implemented. 

The potential ability of source control measures to ensure good IAQ should of course not only reflect the pollution sources indoors but also explored in relation to outdoor air and the ventilation system supplying outdoor air. Outdoor air quality depends on public authorities through their planning actions and building permits and on those responsible for building design and construction, bearing in mind also the actual location of major air pollution sources (industrial or from traffic) in the surroundings having in view the dominant winds directions. The ventilation system can potentially become, itself, an additional source of pollution and therefore should also be the subject to source control procedures.

#### 2.1.3. Definition of Base Ventilation Rate and Decision Diagram to Select the Health-Based Ventilation Rate

As argued above, health-based ventilation guidelines need to refer to “base ventilation rate”, which should create a reference point, a benchmark, defining the limits below which ventilation cannot be reduced without potential consequences to health. The concept of “base ventilation rate” should not only be the reference but it should also motivate additional measures for the control and reduction of pollution sources indoors and outdoors. 

Taking the above into consideration, the base ventilation rate was proposed that is strictly defined as the ventilation required for removing and diluting human bio-effluents when air quality guidelines and exposure limits for all other ambient and indoor pollutants not emitted by humans are satisfied. 

The above definition of base ventilation considers the purely theoretical scenario, in which the only source of indoor air pollution is the occupants. Yet, it is logical and consistent with the general construct of the framework for the health-based ventilation guidelines when air quality guidelines need to be met and source control should be the primary strategy for achieving high IAQ. The concept of base ventilation rates is additionally in agreement with the similar concepts developed in the past. For example, Max von Pettenkoffer, a pioneer of modern hygiene and preventive medicine, proposed a similar concept in the XIX Century [33] while Seifert et al. [25] proposed base ventilation when establishing the principles for defining air quality guidelines and standards.

### 2.2. Determination of the Base Ventilation Rate

HealthVent project proposed that the base ventilation rate be set at 4 L/s per person. The ventilation rate of 4 L/s per person has been defined based on the existing evidence on the effects of human bioeffluents [34,35,36,37,38] and is supported by modelling the typical levels of carbon dioxide (CO_2_) and moisture emitted from humans occurring indoors. This rate corresponds to the minimum ventilation rate prescribed by the standard EN 15251 [9] whenever there are concerns about indoor air quality polluted only by human bio-effluents and is prescribed by the revision of EN 15251 [9] as mentioned in the Introduction section. This rate is also in line with other national and international standards [10,39] and the early guidelines for Nordic building regulations regarding indoor air quality [40]. 

On the other hand, according to epidemiological evidence, a ventilation rate of 4 L/s per person would be insufficient to guard against negative health effects [5,8,41]. For example, a recent review of the scientific literature on the association between ventilation rates and health [8] showed that the lowest ventilation rate at which no negative effects were reported in epidemiological studies was about 6–7 L/s per person. This rate is lower than was identified in earlier reviews, which indicated that a ventilation rate of 10 L/s per person [41] or even 15 L/s per person [5] would be required to avoid any negative health effects. Two reviews suggested that only providing a minimum of 25 L/s per person would eliminate all health risks [6,7]. These reviews used the results of mainly epidemiological studies in buildings and cannot be generalized. The main reason is that it is unclear whether the observed effects were due only to pollutants emitted by humans or included the effects of exposure to other pollutants as well. The levels of indoor pollutants were not properly characterized in the studies reviewed and no information was available on whether exposure reduction in these buildings could be achieved independently of ventilation (e.g., by source control, pollution entrapment, etc.). Although these recommended rates were based on studies that involved a limited number of buildings that were not selected as representative of the general building stock, they do provide a fair indication of the level of ventilation at which no adverse health effects are observed, at least for some health outcomes. 

#### 2.2.1. The Evidence on the Effects of Human Bioeffluents

Humans emit many different volatile organic compounds, which are called human bioeffluents; in addition, they emit moisture and heat [42]. The major human bioeffluent is CO_2_ that is a product of metabolic process occurring in the body; CO_2_ is accepted as a marker of human bioeffluents. There is very little evidence on the effects of human bioeffluents on outcomes other than sensory perception. The recently published studies, also those that examined the effects of major human bioeffluent, namely CO_2_ are summarized in the following. They show that proposed base ventilation rate of 4 L/s per person (corresponding to CO_2_ level of around 1500 ppm, see Section 2.2.2 below) will ensure no elevated risks for health due to exposure to human bioeffluents.

There is no evidence that CO_2_ itself at concentrations occurring indoors is detrimental for health [43,44]. Typical concentrations are well below the occupational threshold limit for CO_2_ of 5000 ppm for an 8-h exposure [45], while the literature shows that levels that are higher than 10,000 ppm evoke negative effects [46]. Some studies have however shown that CO_2_ levels as low as 1000 ppm can produce negative effects on cognitive performance in the form of a reduced ability to make complex decisions [47,48]. These effects may occur due to physiological stress being hazardous for health but so far, there is no proof for this to be the case. One study found additionally that CO_2_ levels of 3000–4000 ppm could affect the ability to perform some office tasks (proofreading) [49]. In contrary, the performance of this task and of other cognitive skills and abilities were not affected at these levels in other studies [43,44,50]. Consequently, the evidence on the effects of CO_2_ on mental abilities and skills can be considered as inconsistent.

There is clear evidence that increased levels of bio-effluents (including CO_2_) do cause a monotonic decrease in the acceptability of air quality and an increase in odour intensity as assessed upon entering a building space polluted by bio-effluents [14,51]. The sensory nuisance caused by the odour produced by bio-effluents is expected to occur at CO_2_ levels down to 500–600 ppm [34], CO_2_ being the marker of human bioeffluents. There was no increase in the intensity of self-reported acute health symptoms at concentration of 1000 ppm considered from Pettenkofer [33] to be a marker of good air quality [35]. No change in the intensity or prevalence of acute health symptoms was observed either when CO_2_ marking the bio-effluents was at 1600 ppm [34] or even at 1800 ppm [36]. At the latter concentration, however, reduction in cognitive performance was observed in the form of a reduced ability to make complex decisions [36]. One study showed that bio-effluents at levels at which CO_2_ concentration was at 2765 ppm increased self-reported sleepiness, heart rate variability and end-tidal CO_2_ concentration [37]; the similar concentration in a different study had negative effects on sleep [38]. Another study showed that bio-effluent levels with CO_2_ at 3000 ppm increased self-reported acute health symptoms including sleepiness, reduced some aspects of cognitive performance and also increased end-tidal CO_2_ concentration and arousal [34,43]. Then they also reduced the ability to perform arithmetical tasks. It can be thus concluded that much higher levels of bioeffluents are needed to produce negative effects other than the nuisance caused by odour or unacceptable air quality.

#### 2.2.2. Modelling the Levels of CO_2_ from Humans

The proposed base ventilation rate of 4 L/s per person will ensure that emissions from humans (human bio-effluents) will be, on average, at the levels at which metabolically produced CO_2_ is below 1500 ppm (Table 2). These levels are much lower than the CO_2_ concentrations of 2500–3000 ppm often observed in residential environments and classrooms; CO_2_ levels higher than 3000 ppm do occur indoors but rather occasionally and for a short duration. This is clearly illustrated in the simulations described below, where different emissions of CO_2_ were examined as a function of human metabolism, while the concentration indoors was estimated as a function of the density of occupation and the outdoor air supply rate. Three specific cases have been considered, namely, simulations were run at 2, 10 and 25 m^2^ per person to model worst-case conditions for different building and space typologies respectively classrooms, offices and residential buildings. 

Table 2 shows that increasing the rate of outdoor air supply (i.e., ventilation rate) lowers the maximum CO_2_ concentration indoors. For a given occupation density, once this CO_2_ level is reached, an equilibrium is established and the concentration does not increase any more. Furthermore, this level is independent of the occupation density. Assuming a typical moderate metabolic rate of 1.2 met, CO_2_ concentrations increase up to a maximum of approximately 1700, 1250 and 1050 ppm at ventilation rates of 4, 6 and 8 L/s per person, respectively.

Occupation densities and ventilation rates also have an impact on the speed of accumulation of CO_2_ indoors. For a particular occupation density, the time it takes to reach a reference CO_2_ level of 1000 ppm is approximately proportional to the ventilation rate. So, for a doubling in ventilation rate a corresponding doubling in the time to reach 1000 ppm indoors is observed. However, the time taken to reach a near-equilibrium level of 98% of the maximum CO_2_ concentration is longer at lower ventilation rates, mainly because the accumulated amount is significantly higher.

Considering the typical profile of occupation periods and density for each building type, the results show that average CO_2_ concentrations range between approximately 1200 and 1500 ppm when the ventilation rate is kept at 4 L/s per person (Table 2, last column). Higher ventilation rates do keep average CO_2_ levels below 1200 ppm and 1000 ppm (respectively for 6 and 8 L/s per person). It should be noted that in particular cases where activities that are more strenuous might occur (i.e., with metabolic rates higher than the 1.2 met level, such as, for example, gymnasiums or industrial settings), higher ventilation rates might be warranted.

#### 2.2.3. Modelling Moisture Levels Produced by Humans

The ventilation rate of 4 L/s per person is expected to keep the relative humidity due to moisture emitted from occupants at levels that avoid any risk for mould growth. For certain climatological conditions this rate will also protect against the proliferation of house dust mites, as confirmed through the simulations of moisture levels indoors, described below, moisture levels being result of moisture release by humans by respiration and sweating. Simulation of moisture generated by activities such as cooking, showering or watering of houseplants by ventilation was not considered, given also that source control through point exhaust is more effective than dilution and removal through ventilation.

Moisture is released from humans both when exhaled air transports some of the water that is lining the upper airways and when it evaporates from the skin, especially when thermal sweating is required to maintain a constant central body temperature. The moisture released by occupants increases the relative humidity levels indoors. While moisture itself is not directly harmful, excessively high levels indoors can adversely affect health, for example, sweating is less effective if air is simultaneously very hot and very humid. 

From the IAQ perspective, only two risks must be considered: (1) the survival and proliferation of house dust mites; and (2) the emergence and development of mould problems. 

To avoid house dust mite infestation, it is generally agreed that the relative humidity of air should be <50% to reduce the mite population and <60% to prevent reproduction [52,53]. To prevent mould growth, it is generally advised that the relative humidity on interior walls, in particular where thermal bridges occur, should be kept below 80% to reduce the risk of condensation [53,54].

Assuming a normal production of water by occupants at low activity levels such as resting or sitting, simple estimations were carried out to determine how much ventilation is required. Three different building typologies (offices/schools, day-care centres and homes) each with typical occupation patterns (8 h/d, 16 h/d, 24 h/d, respectively) and different room dimensions were simulated, as well as two particularly critical ambient conditions in moderate and cold climates (typical for EU):

- Ambient conditions with temperature of −10 °C and a relative humidity of 100%, that is, relatively dry but cold air that could potentially increase the risk of high humidity levels near the inner surfaces of the building envelope;
- Ambient conditions with temperature of +10 °C and a relative humidity of 75% and 85%, representing conditions that can favour the increased risk of house dust mites and mould growth.

Intermediate conditions between −10 °C and +10 °C were simulated, as well.

To avoid the risk of mould growth, using the criteria defined above, the minimum outdoor air supply rates were found to range from 1 to 2 L/s per person. Only under very rarely occurring conditions of high occupation and limited room dimensions were these rates as high as 3 L/s per person.

To avoid the proliferation and reproduction of house dust mites at low ambient temperatures, the minimum outdoor air supply rate was found to be less than 2 L/s per person. With increasing ambient temperatures, the water content of the outdoor air also increased, thus reducing both the effectiveness of controlling humidity through ventilation as well as the ability to control house dust mite proliferation indoors. Consequently, when outdoor relative humidity was below 75%, ventilation rates between 1 and 3 L/s per person were required to avoid the reproduction and proliferation of house dust mites, while as much as 6 L/s per person were required when outdoor relative humidity was 85%. Above this level of ambient relative humidity, ventilation rates from 8 to 13 L/s per person were found to be necessary to reduce the risk of house dust mites.

It should be noticed that, on the one hand, moisture can only be removed by ventilation in conditions where outdoor absolute humidity is lower than indoors and on the other hand, that there are other effective remedial actions to control moisture when that is the case. Thus, since ventilation works through a dilution effect, hot humid climates might require the use of dehumidification strategies/systems to keep indoor relative humidity within the appropriate ranges.

### 2.3. Decision Diagram for Selecting the Health-Based Ventilation Rate for a Specific Building

**Figure 1:**
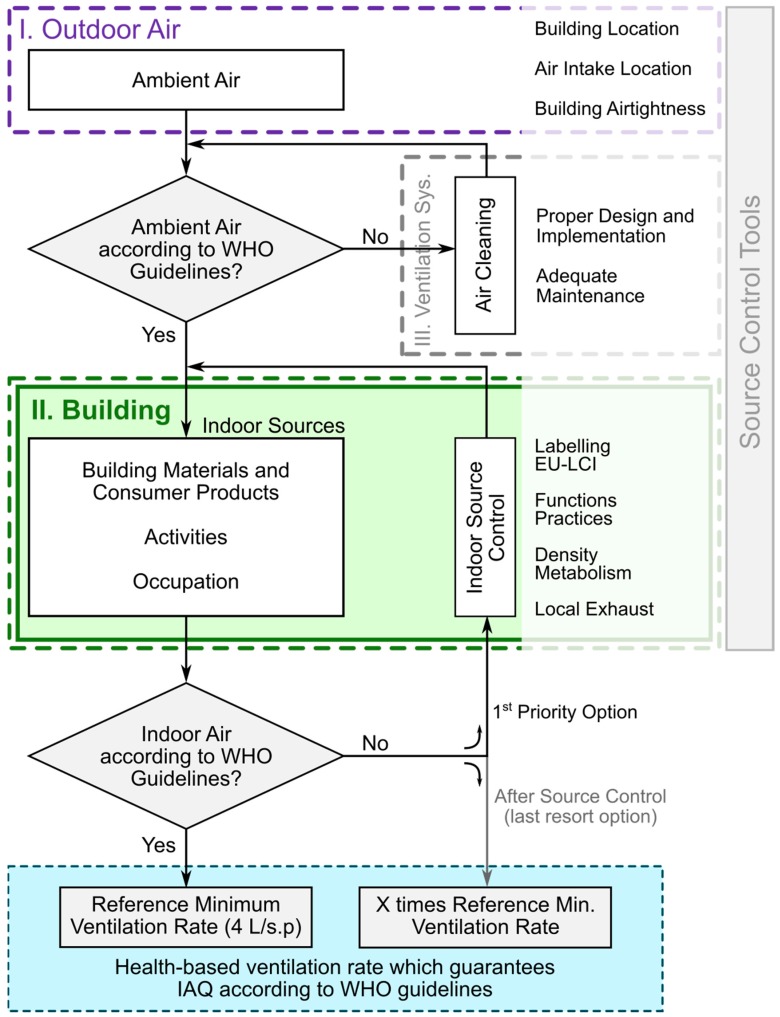
Decision diagram for deriving the adequate health-based ventilation rate for a specific building.

To facilitate the process of determining the specific health-based ventilation rate for a specific building HealthVent project proposed a “decision diagram” which is illustrated in Figure 1. The diagram refers to and respects the three postulates upon which the guidelines are constructed and additionally refers to the construct defining the relationship between outdoor air, building and ventilation (Section 2.1).

The diagram builds on a two-level sequential IAQ management approach in which source control measures are first implemented to their full potential, after which ventilation rates are adjusted for the specific building case, taking into consideration the number and activity level of the occupants. It shows the steps that should be followed and appropriate ways to examine and implement optimal source control strategies at the building design and operational stages and describes how these actions can contribute to properly quantifying the health-based ventilation rate.

The health-based ventilation rate derived using the proposed diagram ensures that WHO air quality guidelines are met and that the requirements regarding base ventilation rate are met as well. The health-based ventilation rate for a specific building will be either equal to or higher than the base ventilation rate.

#### 2.3.1. Outdoor Air

The decision diagram starts with a first checkpoint to verify whether the outdoor air fulfils WHO air quality guidelines. If the air supplied to the building meets the WHO ambient air quality guidelines, then there is no need for special filtration or air cleaning systems. This air can be delivered into the building as is, either by natural or mechanical means, depending on the specific conditions. Clean outdoor air will remove one important potential source of indoor air pollution, the dust trapped in inlet air filters and microbial growth in them.

If the air entering the building does not meet the WHO ambient air quality guidelines, then measures to clean it are required and dedicated equipment that might include continuously operating air filtering and cleaning capabilities should be considered, together with an airtight envelope that isolates occupants from the ambient atmosphere. 

#### 2.3.2. Building

The next step after dealing with outdoor air is to satisfy the requirements of the WHO air quality guidelines at the building level. The building must be designed and built with due consideration of its specific functions and operational practices. For example, there are different demands and requirements for offices, schools and residential buildings. 

Source control of air pollutants is implemented at this stage by national labelling schemes for construction materials and products that are already available in many EU countries. Efforts to develop a harmonization framework for their implementation have been undertaken recently by the European Commission’s Joint Research Centre [55,56,57].

#### 2.3.3. Health-Based Ventilation Requirements

Once all actions aiming at meeting WHO’s air quality guidelines and reducing exposure levels have been exercised, the health-based ventilation rate can be determined. If due respect is given to source control requirements in the building, then the health-based ventilation rate is, by definition, equal to the base ventilation rate. However, whenever the WHO air quality guidelines are not fulfilled, the health-based ventilation requirement is higher than the base rate and is then set as a multiple of the base rate.

#### 2.3.4. Ventilation System

When a dedicated ventilation system is implemented then care must be taken for its proper design, operation and maintenance based on the criteria and values referred above. This should ensure the compliance of the system with health-based ventilation requirements throughout its entire lifetime. This is the only way to avoid health risks due to improper use of a ventilation system in buildings, a situation that was once widespread [58,59] and still continues to be a frequent problem. 

## 3. Benefits from Implementation of the Proposed Framework

The potential benefits of adopting the proposed method for determining health-based ventilation were estimated by examining its effect on the burden of disease. The estimation was made using the model originally developed in the context of the EnVIE project for burden of disease estimations [18]. This model was subsequently updated with the estimated baseline ventilation distributions for European countries [60,61] and a mass-balance based exposure estimation was performed [61,62,63,64]. The results of these simulations estimated that 65% of the total burden of disease was caused by indoor exposures originating from outdoor air. This burden of disease is dominated by cardiovascular diseases (61%) followed by asthma and allergies (18%) and respiratory tract cancers (11%). 

Three hypothetical scenarios were designed to assess the impact of implementing the health-based ventilation guidelines’ framework on population health (Table 3):

- Scenario 1 represents a building stock with simply optimized ventilation (i.e., by changing ventilation only) to minimize the burden of disease.
- Scenarios 2 and 3 represent two alternative ways of improving indoor air quality in buildings. In Scenario 2 it is achieved by enhanced filtration of the outdoor air supplied, to remove ambient pollutants. In Scenario 3 it is attained by source control indoors together with application of a base ventilation rate.

**Figure 2:**
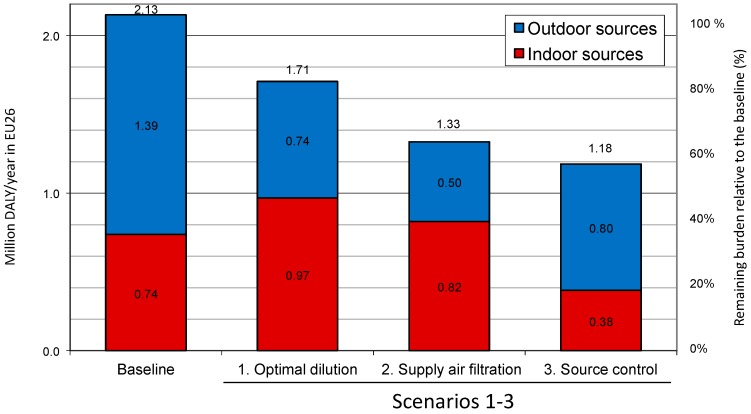
Current and estimated burden of disease in Europe (EU-26, million DALYs/year) for different indoor exposure mitigation approaches (scenarios 1–3, see Table 3 for their definitions).

In Figure 2 the current and estimated burden of disease in Europe (EU-26, million DALYs/year) for the different indoor exposure mitigation approaches are shown.

In the first scenario only a 20% reduction of the burden of disease was achieved because infiltration of outdoor pollution compensates for the reduction of exposures caused by indoor sources at increased ventilation rates; the European (EU-26) minimum burden of disease is achieved at national average ventilation rates from 1 to 9 L/s per person. Typically, the national minimum burden of disease occurs at ventilation rates ranging from about 3 to 5 L/s per person with the EU-26 average being 4.4 L/s per person. 

In the second scenario, substantially larger health gains are projected as the burden of disease was reduced by about 40%. Filtration of the outdoor air allows higher ventilation rates and in this case, the European optimal ventilation rate was achieved at 7.7 L/s per person. In the second scenario, because the WHO guidelines (e.g., for PM_2.5_) are exceeded in areas where 80% of Europeans live [65], filtration of outdoor air would have to be implemented in a majority of buildings. 

In the third scenario, a 55% reduction in the burden of disease was projected. 

The result obtained in the third scenario strongly supports a strategy that combines control of indoor sources by applying a base ventilation rate; such a strategy may have to be supplemented with air filtration, which would bring additional benefits, unless outdoor air quality is regulated at the city level. 

## 4. Discussion

The original intent of the work was to propose health-based ventilation guidelines. However, in the process of delivering this objective it became clear that the project should deliver the framework that is not only limited to the definition of ventilation rates but that is proposing a holistic approach in which all methods for reducing exposures to hazardous pollutants indoors are taken into account. This is clearly demonstrated in the decision diagram (Figure 1), which besides ventilation includes source control, filtration and air cleaning. Furthermore, following the principles of primary prevention, the proposed framework prioritizes the use of source control over ventilation, ventilation being subordinated to source control. The proposed framework should thus be viewed as a framework for exposure control to reduce health risks, in which ventilation requirements are not neglected: they are considered to be important, are defined and included but only once other methods for exposure control are exerted.

It is no surprise that the proposed framework includes the elements already present in the existing guidelines and standards dealing with ventilation and exposure control. It may consequently be asked how the proposed framework is expected to change the quality of air in buildings if the existing regulations were not able to create proper protection. The success of the proposed work is attributed to addressing the issue of poor air quality in a holistic manner as it puts together all strategies and reference guidelines into a systematic, coherent and straightforward structure, where the strategies are placed in orderly manner to achieve the goals considering the resources that are available. Of course, the efficiency of proposed framework has to be documented but there are studies that clearly show that approaches using source control as the primary strategy for reducing exposures are more efficient than simply intensifying the rates at which ventilation of the spaces is achieved.

The proposed decision diagram describes different phases that need to be taken in sequence during definition of actual health-based ventilation rate for a specific building. The diagram imposes that different exposure control strategies other than ventilation should be explored and implemented during the buildings’ design and operational stages at the levels of the outdoor air, the ventilation system and the building itself and its components. Only then the required health-based ventilation rate can be determined. The similar principles have been promoted in the current ventilation standards, especially in the revision of Standard EN15251 [9] called prEN16798-1 [12] which states that the primary method of exposure control should be the reduction of the pollution loads but not that forcefully and unambiguously as in the proposed framework. Declaration of the expected emissions from indoor sources is required by EN 13779 [66], which in addition defines the classes of outdoor air and the air supplied indoors for ventilation in reference to WHO air quality guidelines.

It must be emphasized here that outdoor air brought indoors contributes, along with indoor sources, to the quality of the air to which building occupants must breathe. Clean outdoor air is therefore the first prerequisite for clean air indoors. Consequently, a high priority should be given to tackling air quality at the city level. Consideration should also be given to other aspects, such as the location of the building (e.g., not being placed near highways or roads with heavy traffic or near the source of industrial emissions, paying due attention to the prevailing winds and the topography of the surroundings), the air intake location (e.g., located sufficiently far from chimneys, bus stops or exhaust air outlets) and the overall building airtightness.

The proposed framework stipulates that the health-based ventilation rate cannot be lower than the base ventilation rate once all health exposure guidelines are fulfilled with respect to non-human sources of pollution. In the present framework, this requirement pertains to WHO air quality guidelines. But if other health relevant exposure guidelines are published by cognizant authorities and/or WHO air quality guidelines are updated they should then be integrated within the framework and followed. The base rate is defined to deal with pollutants that are associated with emissions from humans. It should secure no health risks that are associated with exposures to pollutants that are emitted by humans or those that are associated with human emissions (e.g., because of pollutants that are the products of chemical reaction involving pollutants emitted from humans). 

The base ventilation rate creates a true benchmark and reference point for defining ventilation rates based on health criteria by stating that rates lower than this are not allowed. It is set at 4 L/sp considering present evidence on the effects of emission of human bioeffluents on health during normal human activity level. The proposed base ventilation rate of 4 L/sp should be revised if more evidence on the effects of human bio-effluents become available, whether up or down. Hence the base ventilation rate proposed in the framework must be under continuous maintenance. The continuous maintenance applies also to the exposure guidelines, as indicated above.

In case the exposure guidelines cannot be attained by other means than by ventilation, the health-base ventilation rate should be higher than the base rate. The actual level of ventilation or the size of extension above the base rate is determined using the reference guidelines for the pollutant in question and its emission rates, similarly as it is done in the IAQ procedure recommended by Standard 62.1 [10].

## 5. Implementation, Regulations, Research Needs on the Proposed Framework

Implementation of the proposed framework for health-based ventilation guidelines is expected to promote the advance of knowledge and technological innovation and ensure the competitiveness of the European market. At the same time, it is expected that the basic rights stated by the WHO to grow up, live, work and learn in healthy indoor environments will be safeguarded and guaranteed [27,30]. But these can only be achieved if the proposed health-based ventilation guidelines are assisted by the adequate policies supporting their implementation. These actions should ensure that both outdoor and indoor air quality is adequately addressed in all relevant policies, regulations and guidance at international, national, regional and local levels. 

The following tasks are anticipated ensuring the development and implementation of policies, regulations and guidelines:

- A common regulation of ventilation in Europe shall be developed that takes into account local climate and specific aspects of local culture.
- A harmonized framework of construction and consumer products emissions labelling criteria shall be developed.
- Building regulations that require products with certified emissions already at the building design stage shall be developed.
- Adequate regulations shall be developed for indoor air quality maintenance, inspection and operation.
- Criteria and requirements for energy efficient buildings shall be developed in which the requirements for health-based ventilation are decoupled from the requirements placed upon systems for maintaining thermal comfort (heating/cooling).
- European guidance shall be developed on the proper design, construction, maintenance and inspection of ventilation systems.

These tasks should be harmonized and aligned with the already existing or legislative documents and frameworks under development such as:

- Harmonization framework for indoor air monitoring which was recently developed by the European Commission (DG JRC and DG SANCO) in the context of the PILOT INDOOR AIR MONIT project, particularly useful in the context of auditing.
- Harmonization frameworks for indoor products labelling and health-based evaluation which were developed by the European Commission’s Joint Research Centre [56,57], particularly useful in the context of labelling.
- Legislative instruments, such as Ecolabel criteria for various products, Ecodesign Directive Lot 6 on ventilation, CEN/TC 350/WG 5 prEN 16309 “Sustainability of construction works” [67].

One key prerequisite of the proposed framework for health-based ventilation guidelines is that outdoor air quality shall comply with the WHO Air Quality Guidelines and the requirements of the EU Ambient Air Quality Directive in every city and specifically in any future building location area. This puts obvious responsibility on the national, regional and local authorities to keeping the outdoor air clean but also on architects and building designers who should take into account the ambient pollution at the specific building’s location and take appropriate solutions for minimizing it. It is thus postulated that one of the attributes of future energy smart cities should be that the outdoor air is clean and does not create risks for health.

Another important key for the success of the proposed framework is an adequate support and validation by research. The key research areas that consequently need to be addressed in the future include:

- Population-representative measurement campaigns on indoor exposures in all major types of buildings including quantification of ventilation rates and analysis of the health impact of indoor and outdoor sources.
- Investigations that identify the health endpoints that are relevant to indoor exposures particularly with respect to examination of chronic health effects and subpopulations with special needs (vulnerable groups such as children, elderly or people with allergies or other hypersensitivities).

The proposed studies should be holistic and multidisciplinary and should establish correlations between indoor air pollution, ventilation and health effects in relation to thermal comfort and energy efficiency. Exposures to single chemicals and chemical mixtures should be better characterized. Pollutant levels specified in existing and future IAQ guidelines should always be measured. This applies also to measurements of pollutants that may be transported into buildings from outdoors, infiltration parameters and decay rates. Finally, it is crucial that in future studies ventilation measurements that are used to estimate exposures are better characterized and documented. This was not the case in many previous studies [8]. This means that different measuring techniques should be used, ventilation rates should be measured in different seasons and different averaging times should be used. 

## 6. Conclusions

A framework for health-based ventilation guidelines was proposed. The main elements of the framework are as follows:

- Air quality shall comply with WHO Air Quality Guidelines.
- Source control should be the primary strategy for managing indoor air exposures.
- Ventilation is a supplementary strategy for improving indoor air quality in buildings.
- The health-based ventilation rate for a specific building should be determined according to the decision diagram proposed by the HealthVent project.
- The health-based ventilation rate must not be lower than the base ventilation rate.
- The base ventilation rate is proposed to be set at 4 L/s per person.
- Ventilation systems and health-based ventilation standards shall comply with the health-based ventilation guidelines’ framework.

A legislative framework is required to support implementation of the present recommendations. It should identify the key responsibilities of various key stakeholders to ensure that the design, maintenance and operation of buildings comply with the concept and requirements of the framework. 

New research is required to verify the effectiveness and the value of the proposed framework.

## Figures and Tables

**Table 1:** Summary of existing air quality guidelines (the numbers in brackets indicate the averaging time for which the guideline values are applicable).

Pollutant	Air Quality Guidelines		Specific Indoor Air Quality Guidelines	
	AQ WHO (2000)	AQ WHO (2006)	EU-INDEX (2005)	IAQ WHO (2010)
CO (mg/m^3^)	100 (15 min)		100 (15 min)	100 (15 min)
	60 (30 min)		60 (30 min)	60 (30 min)
	30 (1 h)		30 (1 h)	30 (1 h)
	10 (8 h)		10 (8 h)	10 (8 h)
				7 (24 h)
NO_2_ (μg/m^3^)	200 (1 h)	200 (1 h)	200 (1 h)	200 (1 h)
	40 (1 y)	40 (1 y)	40 (1 w)	40 (1 y)
SO_2_ (μg/m^3^)	500 (10 min)	500 (10 min)		
	125 (24 h)	20 (24 h)		
PM_10_ (μg/m^3^)		50 (24 h)		
		20 (1 y)		
PM_2.5_ (μg/m^3^)		25 (24 h)		
		10 (1 y)		
OZONE (μg/m^3^)		100 (8 h)		
RADON (Bq/m^3^)				No safe level Refer. level: 100
Benzene (μg/m^3^)	UR 6 × 10^−6^		No safe level-Not more than outdoor level	No safe level
Tetrachloroethylene (μg/m^3^)	250 (1 y) 8000 (30 m)			250 (1 y)
Toluene (μg/m^3^)	260 (1 w) 1000 (30 m)		300 (long-term)	
Styrene (μg/m^3^)	260 (1 w) 70 (30 m)		250 (long-term)	
Xylenes (μg/m^3^)			200 (long-term)	
Formaldehyde (μg/m^3^)	100 (30 min)		30 (30 min)	100 (30 min)
Naphthalene (μg/m^3^)				10 (1 y)

Abbreviations: m-min, minutes average; h, hour(s) average; w, week average; y, annual average; UR, unit risk-cancer risk estimates for lifetime exposure to a concentration of 1 μg/m^3^; long term, long term exposure.

**Table 2:** CO_2_ concentrations in schools, offices and residential buildings when supplying outdoor air at ventilation rates of 4, 6 or 8 L/s per person (considering activity level of 1.2 met).

Building Type (Occupation Density)	Ventilation Rate (L/s per per.)	CO_2,max_ ppm	Time to Reach 98% of CO_2,max_	Time to 1000 ppm (h:mm)	Typical Occupation Periods	Average CO_2_ ppm
School (2 m^2^/per.)	4	1692	1:31	0:15	5 × 1.5 h classes (20 min. breaks + 1.5 h lunch break)	1456
	6	1261	0:58	0:19		1145
	8	1046	0:42	0:33		977
Office (10 m^2^/per.)	4	1692	7:36	1:18	4 h + 4 h (1.5 h lunch break)	1237
	6	1261	4:54	1:39		1025
	8	1046	3:34	2:45		901
Residential (25 m^2^/per.)	4	1692	19:01	3:15	12 h (continuous)	1182
	6	1261	12:15	4:08		1016
	8	1046	8:55	6:53		904

**Table 3:** Hypothetical scenarios for assessing impacts on burden of disease based on the implementation of the different indoor exposure mitigation approaches.

Hypothetical Scenarios for Assessing Impacts on Burden of Disease Based on the Implementation of the Different Indoor Exposure Mitigation Approaches	
Baseline scenario	Existing building stock and existing distribution of mechanical ventilation by country
Scenario 1	Dilution of indoor emissions by health-based optimisation of national average ventilation rates
Scenario 2	Filtration of particulate matter (PM_2.5_) in the outdoor air supply by 50%
Scenario 3	Source control (90% reduction in second-hand smoke (SHS), carbon monoxide (CO) and radon (Rn); 50% reduction of volatile organic compounds (VOC) and dampness; 25% reduction of indoor generated PM_2.5_) (4 L/s per person) ^1^

^1^ It was assumed that 90% of second hand smoke exposures can be removed or controlled by indoor smoking policies and similarly for radon and carbon monoxide, in these cases by proper construction techniques and source removal. For volatile organic compounds, it was assumed that half of the exposures could be removed by a correct choice of materials, labelling schemes, etc. Dampness and mould problems were assumed to be reduced by 25% using good construction and maintenance practices. All estimated coefficients contain substantial uncertainties.
